# Bibliometrics and National Institutes of Health Funding: Associated Factors in Academic Rhinology

**DOI:** 10.1007/s12070-024-05156-y

**Published:** 2024-10-30

**Authors:** Lucy Revercomb, Aman M. Patel, Om B. Tripathi, David W. Wassef, Paul T. Cowan, Cynthia Schwartz, Andrey Filimonov

**Affiliations:** Department of Otolaryngology–Head and Neck Surgery, Rutgers New Jersey Medical School, 185 S Orange Ave Newark, Newark, NJ 07103 USA

**Keywords:** Research productivity, Bibliometric, Relative citation ratio, NIH funding, Rhinology

## Abstract

Our study aims to evaluate demographics and research productivity in academic rhinology and to establish the relationship between bibliometrics such as the Hirsch index (*h*-index) and the more recently developed relative citation ratio (RCR) and National Institutes of Health (NIH) funding. Retrospective cohort study. The demographics of academic rhinologists were collected from institutional faculty profiles (*N* = 207). Funding data were obtained from the NIH Research Portfolio Online Reporting Tools Expenditures and Reports Database. The *h*-index was calculated using Scopus. The mean (m-RCR) and weighted RCR (w-RCR) were calculated using the NIH iCite tool. The majority of academic rhinologists were men (72.9%). Only 8.7% of rhinologists (*N* = 18) received NIH funding. Rhinologists receiving NIH funding had greater *h*-index (31 vs. 11), m-RCR (2.6 vs. 1.6), and w-RCR (339.9 vs. 44.7) (*P* < 0.001). Men had greater *h*-index (14 vs. 10, *P* < 0.001) and w-RCR (56.8 vs. 36.9, *P* = 0.025) but not m-RCR (1.7 vs. 1.6, *P* = 0.799) than women. Stratifying by academic professorship rank and across all career durations, *h*-index, m-RCR, and w-RCR were not significantly different between men and women. Among academic rhinologists the *h*-index, m-RCR, and w-RCR were all associated with receiving NIH funding. Similar *h*-index, m-RCR, and w-RCR between men and women across all academic professorship ranks and career durations suggests production of similar quality and quantity of research. The m-RCR and w-RCR help to address some of the limitations of the *h*-index and are useful for assessing research productivity.

## Introduction

Assessing research productivity with bibliometrics is important in the determination of research funding allocation, promotion, and tenure [1–4]. There are benefits and detriments to each bibliometric and it stands to be determined which provides the best approximation of research productivity. The Hirsch index (*h*-index) remains one of the most commonly utilized bibliometrics [3]. An author’s *h*-index is calculated by the *h* number of published papers with at least *h* citations each. This calculation allows the *h*-index to relay both quality and quantity of an author’s research. However, the dependence on quantity of publications and citations limits comparison of physicians at different career stages and comparison between larger academic fields with more practitioners and readership and smaller fields like rhinology.

Although the *h*-index effectively captures certain aspects of research productivity, to address its limitations the National Institutes of Health (NIH) developed the relative citation ratio (RCR) [5]. Each publication by an author receives an RCR, calculated as the total citations received by a publication per year divided by the average number of citations received per year by NIH-funded articles in the same field. To determine articles in the same field, the RCR utilizes a co-citation network, which predicts the presence of a distinct academic community by identifying groups of articles that cite one another [6]. The RCR from each article can be averaged to calculate the mean RCR (m-RCR) or summed to calculate the weighted RCR (w-RCR). The m-RCR, as an average, is independent of publication totals and quantifies scientific impact. The w-RCR, as a sum, provides a measure of research productivity over time. The RCR co-citation network normalizes different fields and allows comparison between different scientific disciplines. It is important to evaluate the RCR in different fields because of this distinct co-citation network, and benchmark data in academic rhinology are currently lacking.

Otolaryngology projects account for a significant amount of NIH funding, ranking fourth of eight among surgical subspecialties for total funding between 2009 and 2019. Rhinology accounted for 2.5% of NIH-funded otolaryngology research projects during this time period, with mostly basic science studies (63.6%) [7]. Bibliometrics can be utilized by institutions for determinations of promotions and tenure and by physicians to understand the relationship between research productivity and receiving NIH funding. Although previous studies have demonstrated an association between the *h*-index and NIH funding in otolaryngology, the association in rhinology between the RCR and receiving NIH funding remains unclear [2]. Assessing a cohort of 207 academic rhinologists in the United States, our study aims to evaluate the association between bibliometrics, receiving NIH funding, and the research productivity of rhinologists as measured by the *h*-index, m-RCR, and w-RCR.

## Methods

### Study Population

Academic rhinologists were identified through faculty listings published on the websites of all otolaryngology residency and rhinology fellowship programs in the United States. Otolaryngology residency programs were obtained from the Fellowship and Residency Electronic Interactive Database Access, published by the American Medical Association [8]. Rhinology fellowship programs recognized by the Accreditation Council for Graduate Medical Education were obtained from listings published by the American Rhinologic Society [9]. Information collected included gender, master’s degree status, PhD status, academic rank (assistant, associate, full professor, or hospital-employed without professorship), career duration, and current location. Gender was determined by the name, photographs, and pronouns provided on publicly available academic profiles. Career duration was determined by subtracting the year of a rhinologist’s medical school graduation from 2024, the year of biographic data collection. Current location was defined by U.S. Census Bureau designations. Emeriti, adjunct, visiting, part-time, and non-academic clinical faculty were excluded. The Rutgers New Jersey Medical School Institutional Review Board exempted our study because of the public, de-identified nature of the data.

### NIH Funding

**Fig. 1:**
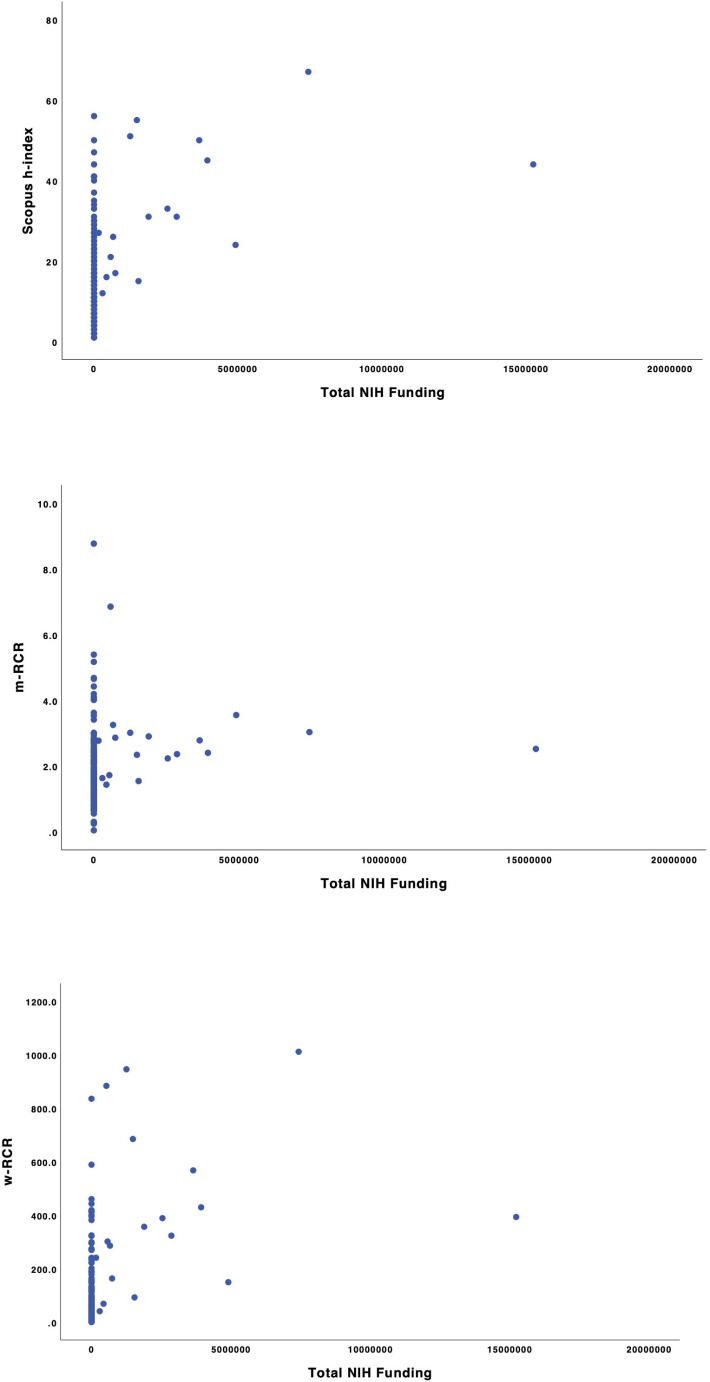
(**a**) Scopus h-index by National Institutes of Health (NIH) funding among 207 rhinologists (*R* = 0.330). (**b**) Mean-relative citation ratio (m-RCR) by NIH funding among 207 rhinologists (*R* = 0.144). (**c**) Weighted-relative citation ratio (w-RCR) by NIH funding among 207 rhinologists (*R* = 0.396)

The funding status of each academic rhinologist was determined using the NIH Research Portfolio Online Reporting Tools Expenditures and Reports (RePORTER) website (https://reporter.nih.gov/) (Fig. 1). NIH funding received between January 1985 and December 2023 was recorded. We collected total lifetime NIH funding including research grants (R series), career development awards (K series), research training and fellowships (T & F Series), program project/center grants (P series), resource grants, and trans-NIH programs. NIH funding data were collected in March 2024.

### Calculation of Bibliometrics

The *h*-index was calculated for each rhinologist using the Scopus Database calculator (https://www-.scopus.com/search/form.uri? display = basic#author). The m-RCR and w-RCR were calculated using the NIH iCite tool (https://icite.od.nih.gov/). To account for discrepancies in search results based on author name in iCite, the Scopus author lookup feature was utilized to export PubMed IDs from an author’s profile into iCite to record m-RCR and w-RCR, as previously described [10, 11].

### Statistical Analysis

The chi-square and Kruskal-Wallis tests were implemented to compare categorical and continuous variables, respectively, as the continuous data were found to be non-parametric. The two-sided threshold for statistical significance was set at *P* < 0.05 for all statistical tests. SPSS version 25 (IBM) was utilized for all statistical analysis.

## Results

In total, 207 academic rhinologists satisfied inclusion criteria. The majority were men (72.9%), assistant professors (36.7%), and without a master’s degree (89.4%) or PhD (95.7%) (Table 1). A small minority (8.7%) received NIH funding. Rhinologists receiving NIH funding were more likely to have a PhD degree (27.8% vs. 2.1%, *P* < 0.001), full professor status (50.0% vs. 15.9%, *P* = 0.002), and career duration ≥ 21 years (44.4% vs. 24.9%, *P* = 0.045) than those not receiving NIH funding. Rhinologists receiving NIH funding had significantly greater median (interquartile range [IQR]) *h*-index (31 [20–50] vs. 11 [7–18]), m-RCR (2.6 [2.1-3.0] vs. 1.6 [1.1–2.2]), and w-RCR (339.9 [159.8-596.8] vs. 44.7 [18.4–107.0]) than those not receiving NIH funding (*P* < 0.001).

**Table 1:** Rhinologist demographics and research productivity by receipt of NIH funding, n (%)

	No NIH funding	NIH funding	*P* value	Total
No.	189 (91.3)	18 (8.7)	-	207 (100.0)
Gender				
Man	136 (72.0)	15 (83.3)	0.299	151 (72.9)
Woman	53 (28.0)	3 (16.7)		56 (27.1)
Master’s degree				
No	169 (89.4)	16 (88.9)	0.945	185 (89.4)
Yes	20 (10.6)	2 (11.1)		22 (10.6)
PhD degree				
No	185 (97.9)	13 (72.2)	**< 0.001**	198 (95.7)
Yes	4 (2.1)	5 (27.8)		9 (4.3)
Academic rank				
Assistant professor	73 (38.6)	3 (16.7)	**0.002**	76 (36.7)
Associate professor	55 (29.1)	6 (33.3)		61 (29.5)
Full professor	30 (15.9)	9 (50.0)		39 (18.8)
Hospital-employed without professorship	31 (16.4)	0 (0.0)		31 (15.0)
Career duration				
0–10 years	40 (21.2)	0 (0.0)	**0.045**	40 (19.3)
11–20 years	102 (54.0)	10 (55.6)		112 (54.1)
$\:\ge\:$ 21 years	47 (24.9)	8 (44.4)		55 (26.6)
Current location				
Northeast	49 (25.9)	4 (22.2)	0.446	53 (25.6)
Midwest	48 (25.4)	2 (11.1)		50 (24.2)
South	57 (30.2)	8 (44.4)		65 (31.4)
West	35 (18.5)	4 (22.2)		39 (18.8)
Research productivity, median (IQR)				
h-index	11 (7–18)	31 (20–50)	**< 0.001**	13 (7–21)
m-RCR	1.6 (1.1–2.2)	2.6 (2.1-3.0)	**< 0.001**	1.7 (1.2–2.3)
w-RCR	44.7 (18.4–107.0)	339.9 (159.8-596.8)	**< 0.001**	52.1 (19.2-130.1)

Abbreviations IQR, interquartile range; m-RCR, mean relative citation ratio; NIH, National Institutes of Health; w-RCR, weighted relative citation ratio

Overall rhinologists received $50,140,030 in NIH funding from 1985 to 2023 (Table 2). The median (IQR) NIH funding received was $1,513,832 ($565,289-3,719,359) and did not vary by gender, master’s degree, PhD degree, career duration, or current location. Full professors receiving NIH funding received greater median funding than associate professors ($3,648,506 vs. $1,059,353, *P* = 0.037). w-RCR had the highest correlation with NIH funding quantity (*R* = 0.396) versus *h*-index (*R* = 0.330) and m-RCR (*R* = 0.144) (Fig. 1).

**Table 2:** NIH funding among 18 rhinologists receiving it by demographics

	NIH funding ($)	Median (IQR)	*P* value
Total	50,140,030	1,513,832 (565,289-3,719,359)	-
Gender			
Man	47,727,889	1,890,373 (662,548-3,931,917)	0.173
Woman	2,412,141	575,619	
Master’s degree			
No	42,414,172	1,513,833 (597,351-3,452,942)	0.999
Yes	7,725,858	3,862,929	
PhD degree			
No	41,778,865	1,543,086 (554,959-3,790,212)	0.522
Yes	8,361,165	738,330 (412,159-3,399,259)	
Academic rank			
Assistant professor	1,694,314	662,548	**0.037**
Associate professor	7,148,914	1,059,353 (364,255-2,054,359)	
Full professor	41,296,802	3,648,506 (1,369,178-6,170,283)	
Hospital-employed without professorship	-	-	
Career duration (years)			
0–10	-	-	0.051
11–20	13,467,609	700,439 (397,172-2,626,299)	
$$\:\ge\:$$ 21	36,672,421	2,911,145 (1,311,478-6,801,353)	
Current location			
Northeast	5,431,773	1,111,455 (681,494-2,280,882)	0.925
Midwest	4,409,334	2,204,667	
South	29,968,759	2,451,142 (353,652-4,664,087)	
West	10,330,164	1,232,996 (467,717-6,046,910)	

Abbreviations IQR, interquartile range; NIH, National Institutes of Health

Men had greater *h*-index (14 [8–24] vs. 10 [5–16], *P* = 0.004) and w-RCR (56.8 [19.9-152.5] vs. 36.9 [9.2–94.3], *P* = 0.025) than women (Table 3). Men and women did not have significantly different m-RCRs (1.7 [1.2–2.3] vs. 1.6 [1.1–2.4], *P* = 0.799). Men were associated with higher academic rank (*P* = 0.021), with 22.5% of men with full professor status vs. 8.9% of women. Men and women did not significantly differ for master’s degree status, PhD status, or career duration.

**Table 3:** Rhinologist demographics and research productivity by gender, n (%)

	Men	Women	*P* value
No.	151 (72.9%)	56 (27.1%)	-
Master’s degree			
No	134 (88.7)	51 (91.1)	0.629
Yes	17 (11.3)	5 (8.9)	
PhD degree			
No	143 (94.7)	55 (98.2)	0.271
Yes	8 (5.3)	1 (1.8)	
Academic rank			
Assistant professor	48 (31.8)	28 (50.0)	**0.021**
Associate professor	43 (28.5)	18 (32.1)	
Full professor	34 (22.5)	5 (8.9)	
Hospital-employed without professorship	26 (17.2)	5 (8.9)	
Career duration			
0–10 years	26 (17.2)	14 (25.0)	0.261
11–20 years	81 (53.6)	31 (55.4)	
$\:\ge\:$ 21 years	44 (29.1)	11 (19.6)	
Current location			
Northeast	41 (27.2)	12 (21.4)	0.754
Midwest	34 (22.5)	16 (28.6)	
South	48 (31.8)	17 (30.4)	
West	28 (18.5)	11 (19.6)	
Research productivity, median (IQR)			
h-index	14 (8–24)	10 (5–16)	**0.004**
m-RCR	1.7 (1.2–2.3)	1.6 (1.1–2.4)	0.799
w-RCR	56.8 (19.9-152.5)	36.9 (9.2–94.3)	**0.025**

Abbreviations IQR, interquartile range; m-RCR, mean relative citation ratio; w-RCR, weighted relative citation ratio

Academic rhinologists with a PhD had higher *h*-index (27 [21–29] vs. 12 [7–20], *P* = 0.004), m-RCR (2.9 [2.7–3.1] vs. 1.6 [1.1–2.2], *P <* 0.001) and w-RCR (285.7 [156.3–369.0] vs. 47.4 [15.9-121.1], *P* = 0.001) than those without a PhD (Table 4). Higher academic rank was associated with higher *h*-index, m-RCR, and w-RCR (*P* < 0.001). Full professors had greater *h*-index (30 [23–44] vs. 15 [10–20]), m-RCR (2.3 [1.8–2.8] vs. 1.6 [1.1–2.3]), and w-RCR (270.4 [121.8-429.3] vs. 30.2 [63.9-125.2]) than associate professors (*P* < 0.001). Career duration was also significantly associated with *h*-index, m-RCR, and w-RCR (*P* < 0.001).

**Table 4:** Research productivity by rhinologist demographics, median (IQR)

	h-index	*P* value	m-RCR	*P* value	w-RCR	*P* value
Gender						
Man	14 (8–24)	**0.004**	1.7 (1.2–2.3)	0.799	56.8 (19.9-152.5)	**0.025**
Woman	10 (5–16)		1.6 (1.1–2.4)		36.9 (9.2–94.3)	
Master’s degree						
No	12 (7–20)	0.055	1.6 (1.2–2.3)	0.309	48.0 (18.4-127.7)	0.113
Yes	16 (11–28)		1.9 (1.2–2.5)		87.8 (22.0-200.0)	
PhD degree						
No	12 (7–20)	**0.004**	1.6 (1.2–2.2)	**< 0.001**	47.4 (15.9 -121.1)	**0.001**
Yes	27 (21–29)		2.9 (2.7–3.1)		285.7 (156.3–369.0)	
Academic rank						
Assistant professor	9 (5–12)	**< 0.001**	1.6 (1.1–2.2)	**< 0.001**	28.7 (9.9–61.0)	**< 0.001**
Associate professor	15 (10–20)		1.6 (1.1–2.3)		30.2 (63.9-125.2)	
Full professor	30 (23–44)		2.3 (1.8–2.8)		270.4 (121.8-429.3)	
Hospital-employed without professorship	7 (5–15)		1.6 (0.9–1.9)		18.9 (5.4–56.8)	
Career duration (years)						
0–10	7 (5–9)	**< 0.001**	1.5 (1.1–1.9)	**< 0.001**	19.2 (9.8–35.2)	**< 0.001**
11–20	12 (7–16)		1.6 (1.1–2.3)		45.9 (19.5–99.7)	
$\:\ge\:$ 21	27 (17–41)		2.1 (1.7–2.7)		150.5 (64.5-324.5)	
Current location						
Northeast	11 (6–17)	0.094	1.5 (1.1–2.1)	**0.037**	30.8 (20.5–98.5)	0.057
Midwest	13 (8–20)		1.7 (1.3–2.1)		48.2 (20.5–98.5)	
South	12 (7–27)		1.7 (1.1–2.5)		44.7 (18.0-165.1)	
West	16 (11–22)		1.9 (1.4–2.9)		70.8 (42.5-233.9)	

Abbreviations IQR, interquartile range; m-RCR, mean relative citation ratio; w-RCR, weighted relative citation ratio

On bibliometric analysis stratified by gender and academic rank, among assistant, associate, and full professors *h*-index, m-RCR, and w-RCR were not significantly different (Table 5). Among hospital-employed rhinologists without professorship men had significantly greater *h*-index than women (8 [5–16] vs. 3 [2–8], *P* = 0.041), but not m-RCR (1.6 [1.1-2.0] vs. 1.4 [0.8–1.8], *P* = 0.485) or w-RCR (19.2 [9.1–67.2] vs. 7.4 [4.6–33.1], *P* = 0.133). When stratified by gender and career duration, across all durations *h*-index, m-RCR, and w-RCR were not significantly different between men and women (Table 6).

**Table 5:** Research productivity by rhinologist gender and academic rank, median (IQR)

	No. (%)	h-index	*P* value	m-RCR	*P* value	w-RCR	*P* value
Assistant professor							
Man	48 (63.2)	9 (6–13)	0.307	1.6 (1.1–2.2)	0.511	30.5 (18.1–61.8)	0.129
Woman	28 (36.8)	7 (4–12)		1.5 (1.0-2.2)		19.2 (6.8–44.0)	
Associate professor							
Man	43 (70.5)	15 (11–19)	0.470	1.5 (1.1–2.1)	0.195	62.3 (30.4-152.5)	0.912
Woman	18 (29.5)	15 (9–20)		1.7 (1.2–2.8)		76.4 (28.4-105.7)	
Full professor							
Man	34 (87.2)	30 (23–46)	0.570	2.2 (1.8–2.8)	0.193	254.8 (114.2-447.2)	0.966
Woman	5 (12.8)	29 (24–34)		2.5 (2.2–3.5)		295.6 (156.7-370.5)	
Hospital-employed without professorship							
Man	26 (83.9)	8 (5–16)	**0.041**	1.6 (1.1-2.0)	0.485	19.2 (9.1–67.2)	0.133
Woman	5 (16.1)	3 (2–8)		1.4 (0.8–1.8)		7.4 (4.6–33.1)	

Abbreviations IQR, interquartile range; m-RCR, mean relative citation ratio; w-RCR, weighted relative citation ratio

**Table 6:** Research productivity by rhinologist gender and career duration, median (IQR)

	No. (%)	h-index	*P* value	m-RCR	*P* value	w-RCR	*P* value
0–10 years							
Man	26 (65.0)	6 (5–9)	0.512	1.5 (1.2–1.9)	0.590	19.2 (13.0-34.9)	0.671
Woman	14 (35.0)	7 (4–11)		1.5 (0.8-2.0)		17.0 (6.2–40.6)	
11–20 years							
Man	81 (72.3)	13 (8–17)	0.053	1.6 (1.1–2.3)	0.881	52.1 (21.3-121.3)	0.153
Woman	31 (27.7)	9 (5–15)		1.5 (1.1–2.5)		40.3 (8.2–87.7)	
$\:\ge\:$ 21 years							
Man	44 (80.0)	28 (18–41)	0.055	2.0 (1.7–2.5)	0.349	151.4 (64.6–384.0)	0.366
Woman	11 (20.0)	21 (10–33)		2.4 (1.7–2.8)		118.6 (30.1-295.6)	

Abbreviations IQR, interquartile range; m-RCR, mean relative citation ratio; w-RCR, weighted relative citation ratio

## Discussion

The NIH is the leading source for biomedical research funding in the United States, and receiving NIH funding is a highly competitive and prestigious process [2, 11–13]. For institutions hiring and promoting researchers, and academic rhinologists hoping to obtain NIH funding, understanding factors associated with receiving NIH funding is important. Among the otolaryngology subspecialties, rhinology consistently ranks highly for research productivity, with recent studies showing only head and neck surgery and otology with greater productivity as measured by *h*-index [14, 15]. Our study found a mean *h*-index of 16.5 among 207 academic rhinologists, increased from an average *h*-index of 8.6 reported in 2013 and of 12.4 in 2019 [4, 15]. The continual rise in average *h*-index likely reflects a trend in increased clinical research to advance the field of rhinology. In comparison to other surgical specialties, a 2018 study reported average *h*-indices of 12.0, 13.1, and 18.7 among plastic, general, and thoracic surgeons, respectively [16]. Despite a continuously demonstrated high research productivity, only 8.7% of rhinologists received NIH funding. Additionally, only 2.5% of NIH-funded otolaryngology research projects between 2009 and 2019 were within rhinology [11]. The minority of rhinologists receiving NIH funding may represent a call to action because NIH funding has been shown to play a critical role in advancing science and improving patient outcomes. Academic rhinologists are uniquely positioned to conduct impactful research because of their access to hospital and university resources, and should be encouraged to apply for NIH funding. Institutions should also aim to offer more support for rhinologists seeking NIH funding.

A strong association between *h*-index and receipt of NIH funding in otolaryngology has been documented, but the association of m-RCR and w-RCR with receiving NIH funding in academic rhinology is yet to be established [2]. Our study similarly found academic rhinologists receiving NIH funding had significantly higher median *h*-index than those not receiving funding. There additionally was a significant difference in m-RCR and w-RCR between those receiving NIH funding and their non-NIH funded peers. The association of *h*-index, m-RCR, and w-RCR likely represents the importance of both quality and quantity of research to receive NIH funding. In turn it also likely reflects the high quality and quantity of research output by those who receive NIH funding. Academic rank and career duration were also associated with NIH funding. This relationship highlights the importance of research experience in identifying research questions, applying for funding, and producing impactful research. Academic rank was also the only factor significantly associated with quantity of NIH funding, possibly demonstrating the importance of experience acquired throughout one’s career duration in obtaining NIH funding and additionally the potential for NIH funding to accelerate academic promotion.

Only 27.1% of rhinologists in our study were women, demonstrating underrepresentation of women in rhinology despite efforts to enhance inclusivity [17–20]. Additionally, only 12.8% of full professors were women. Assessing gender disparities in research productivity and bibliometrics is necessary to better understand the paucity of women in academic rhinology leadership positions, as research productivity is paramount to determining academic promotion [21–24]. Overall men had significantly greater *h*-index and w-RCR, but not m-RCR than women. The m-RCR as an average and thus independent of the total number of publications, is the most direct reflection of the quality of research. The similarity of m-RCR between men and women suggests the production of similarly impactful research. The difference in *h*-index and w-RCR therefore likely reflects a difference in the quantity of research being produced by men and women. This may be attributed to shorter career durations among women as 29.1% of men but only 19.6% of women had a career duration $\:\ge\:$ 21 years. With increasing representation of women in rhinology differences between men and women in bibliometrics reflecting quantity of research, such as *h*-index and w-RCR, may be observed to decrease.

Although initial studies in otolaryngology demonstrated disparities in research productivity between early career men and women otolaryngologists that diminished throughout their careers, a more recent study showed initially similar *h*-index scores between men and women with a widening gap as surgeons progressed through their careers [25–27]. To assess differences between men and women throughout career duration and academic rank, we stratified research productivity by these factors. Our study found that when stratified by career duration there were no significant differences between men and women for *h*-index, m-RCR, or w-RCR regardless of career duration or professor rank. The similarity of these three bibliometrics between men and women at equal career stages demonstrates production of similar quality and quantity of research and that disparities in research productivity are diminishing [28]. The only significant difference found was for *h*-index between hospital-employed men and women without professorship. This academic rank had the lowest measures of research productivity overall and the difference between men and women may reflect lower research interests among those without professorship with the additional potential that those without professorship are not provided the support necessary for research.

Our study found a significant association between the *h*-index, m-RCR, and w-RCR and both academic rank and career duration. The difference in not just w-RCR, which as a summation is greatly influenced by career duration, but also in m-RCR, reflects the value of experience in conducting research. This aligns with studies in ophthalmology, neurosurgery, and radiation oncology which similarly found an association between both m-RCR and w-RCR and increased career duration [29–31]. One goal of the RCR, and the m-RCR specifically, is to decrease the time dependence of bibliometrics for comparison of researchers in different phases of their career. Although the increase in m-RCR with career duration may be attributed to experience, it is also important to consider that the m-RCR still maintains some time dependence due to the inherent increase in number of citations that occur for a publication over time.

The relationship between PhD degree status and research productivity has been reported in various subspecialties [6, 29]. Our study showed significantly greater *h*-index, m-RCR, and w-RCR when comparing those with and without a PhD. Those receiving NIH funding were also significantly more likely to have a PhD degree than those without NIH funding. Studies in neurosurgery and orthopedic surgery similarly demonstrated significantly greater m-RCR and w-RCR in those with a PhD [6, 32]. The years of training devoted to research, often with the goal of more significant research involvement, likely explains the higher bibliometrics in those with PhDs [29–32]. Otolaryngologists with a PhD may be more likely to have dedicated research time or run a basic science lab as well. Our study showed no significant association between master’s degree and *h*-index, m-RCR, and w-RCR, potentially due to the difference in level of training and commitment to research between a master’s and PhD degree.

Our previous study of research productivity in academic otology found that those with NIH funding were more likely to have a master’s degree, PhD degree, or higher academic rank [9]. Our present study of research productivity in rhinology shows that PhD degree and higher academic rank were associated with NIH funding, but not master’s degree status. The greater research dedication of a PhD and the greater experience that comes with higher academic rank likely explains the consistency of these findings across otology and rhinology. Of note, both in otology and rhinology, those with NIH funding had significantly greater *h-*index, m-RCR, and w-RCR, demonstrating the association of these bibliometrics with NIH funding across different specialties. Interestingly, both in otology and rhinology, *h*-index and w-RCR, but not m-RCR, were significantly greater for men than women. This continuity demonstrates the comparable quality of research being produced by men and women across different otolaryngology subspecialties. Accordingly, for those evaluating applicants for grants or academic promotion m-RCR may be a better metric as it may display less gender bias. Compared to no significant differences in *h*-index, m-RCR, or w-RCR in rhinology, in otology, only across higher academic ranks and longer career durations did men and women have a similar *h*-index, m-RCR, or w-RCR. These differences may be attributed to variations in the timelines of increased representation of women in these specialties, and in the future, similarities in research productivity across all career durations and academic ranks in otology may occur, as was seen in rhinology.

Several limitations in our study warrant acknowledgement. Academic rank was defined by position at the time of data collection and does not account for career changes between non-academic and academic positions. Limitations of the *h*-index including lack of accounting for author contribution, time since publication, and difficulty comparing among fields and career stages, remain a factor. Differing values may have been calculated if an *h*-index calculator other than Scopus had been used. The RCR also maintains its own limitations in reporting research productivity. Similar to the *h*-index, it cannot account for author order or the inherent increase in citations that occurs with increased time since publication. A 2-year latency period is also required by the iCite database for field-normalization, which may underestimate some authors’ RCRs. This field normalization relies upon the co-citation network of the RCR and although there are an increasing number of studies validating its use in various scientific fields, future studies are still warranted [6, 9, 30–32]. The bibliometrics in this study are derived from citations which themselves are an imperfect metric of quality. One such limitation is the occurrence of selective self-citation or citation of peers, with a recent study also showing gender alignment between citing and cited authors and accordingly fewer citations of publications with female lead authors versus those led by males [33]. Additionally, there are many research funding opportunities outside of the NIH that were not accounted for in this analysis [34, 35]. Our study also does not account for the rhinologists conducting research outside of an academic institution, who still produce meaningful and impactful research. We are unable to assess the time-dependent relationship between research productivity and NIH funding, and whether increased research productivity directly resulted in the acquisition of NIH funding. Future studies should explore the time-dependent relationship of these factors. Our study is limited by small sample sizes which may have precluded the detection of statistically significant differences, especially between men and women. Bibliometrics are only one metric for assessing one’s career and should be evaluated in the context of a holistic view of a physician’s accomplishments throughout his or her career.

## Conclusion

The m-RCR and w-RCR help to address some of the limitations of the *h*-index and are useful for assessing research productivity. Higher *h*-index, m-RCR, and w-RCR were all associated with receiving NIH funding emphasizing the importance of research productivity in both quality and quantity in obtaining NIH funding. Academic rhinologists demonstrated high metrics of research productivity and more NIH funding in the field may enhance the impact of these researchers on patient outcomes. When stratified by career duration and academic rank, men and women did not significantly differ in research productivity as measured by *h*-index, m-RCR, and w-RCR. These similarities in research productivity between men and women hopefully demonstrate a decrease in disparities and a future increase in the representation of women in rhinology.
